# Enhancing Crystallinity and Orientation by Hot-Stretching to Improve the Mechanical Properties of Electrospun Partially Aligned Polyacrylonitrile (PAN) Nanocomposites

**DOI:** 10.3390/ma4040621

**Published:** 2011-04-06

**Authors:** Zhenyu Song, Xiaoxiao Hou, Liqun Zhang, Sizhu Wu

**Affiliations:** 1 Key Laboratory of Carbon Fiber and Functional Polymers, Ministry of Education, College of Materials Science & Engineering, Beijing University of Chemical Technology, Beijing 100029, China; E-Mails: key200511045@163.com (Z.S.); zhanglq@mail.buct.edu.cn (L.Z.); 2 Institute of Coal Chemistry, Chinese Academy of Science, Taiyuan 030001, China; E-Mail: hxx198525@yahoo.cn

**Keywords:** electrospinning, polyacrylonitrile, single-walled carbon nanotubes, nanocomposites

## Abstract

Partially aligned polyacrylonitrile (PAN)-based nanofibers were electrospun from PAN and PAN/single-walled carbon nanotubes (SWNTs) in a solution of dimethylformamide (DMF) to make the nanofiber composites. The as-spun nanofibers were then hot-stretched in the oven to enhance its orientation and crystallinity. With the introduction of SWNTs and by the hot-stretched process, the mechanical properties will be enhanced correspondingly. Scanning electron microscopy (SEM), transmission electron microscopy (TEM), *X*-ray scattering (XRD), differential scanning calorimetry (DSC), and the tensile test were used to characterize the microstructure and performances of the nanofibers. The orientation and crystallinity of the as-spun and hot-stretched nanofibers confirmed by *X*-ray have increased. Differential scanning calorimetry showed that the glass transition temperature of PAN increased about 3 °C by an addition of 0.75 wt% SWNTs indicating a strong interfacial interaction between PAN and SWNTs. The tensile strength and the modulus of the nanofibers increased revealing significant load transfer across the nanotube-matrix interface. For PAN nanofibers, the improved fiber alignment, orientation and crystallinity resulted in enhanced mechanical properties, such as the tensile strength and modulus of the nanofibers. It was concluded that the hot-stretched nanofiber and the PAN/SWNTs nanofibers can be used as a potential precursor to produce high-performance nanocomposites.

## 1. Introduction

Carbon nanotubes (CNTs) have an intriguing application due to their appealing mechanical [1,2], electrical [3], and thermal conductivity properties [4]. In particular, their exceptional mechanical properties and characteristic cylindrical structures with high aspect ratio make them an ideal reinforcing material for polymer nanofibers [5,6,7,8]. However, before full realization of their reinforcing improvement, the following two crucial issues have to be solved: (i) dispersion and orientation of inorganic particles in the nanofiber [9,10], good interfacial bonding is required to achieve load transfer across the SWNTs-matrix interface [11,12]; (ii) the macroscopic alignment in the nanofibers [13], the orientation and crystallinity of polymer chains.

Polyacrylonitrile (PAN) nanofiber is a common precursor of general carbon fibers. However, these applications of PAN nanofibers may be hindered by the poor strength, attributed to their small diameters and unoptimized molecular orientation and crystallinity in the fibers [14]. An alternative promising avenue to produce CNTs-reinforced PAN nanofibers is electrospinning, a method commonly used to create new nanocomposite materials based on organic and inorganic fillers [4]. One advantage of electrospinning is that CNTs orient parallel to the main nanofiber axis during nanofiber formation, on condition that the CNTs are well dispersed in the initial solution [15].

To enhance the strength of the nanofibers, the inorganic single-walled carbon nanotubes (SWNTs) has been considered as the ideal reinforced materials due to their excellent mechanical properties, good electrical and thermal conductivity [16]. Also, stretching of the composites can improve the mechanical properties of polymer nanofibers [17]. Some researchers tried hot-stretched to improve the molecular orientation and the crystallinity of the nanofibers [18,19,20,21]. Therefore, in the work presented here, PAN nanofibers containing SWNTs with concentration 0 wt%, 0.25 wt%, 0.5 wt%, 0.75 wt%, 1 wt% were produced, and then hot-stretched which demonstrated good dispersion of SWNTs and with high orientation and crystallinity of PAN molecules.

## 2. Experimental Section

### 2.1. Materials

PAN used in this study included PAN/methyl acrylate/itaconic acid (93:5.3:1.7 w/w) (average molecular weight of 100,000 g/mol) which was purchased from UK Courtaulds Ltd. The solvent was *N*, *N*-dimethylformamide (DMF, Beijing Chemical Plant Co.). In order to disperse the SWNTs in the organic polymer matrix in uniformity, the SWNTs were modified to form an individually polymer-wrapped structure [22]. As is well known, polymer wrapped greatly inhibits the Van der Waals attraction between the polymer with solvent and the interactional polymer chains which normally observed between separate SWNTs with small ropes of SWNTs. These effects caused the wrapped nanotubes to be much more readily suspended in concentrated SWNTs solutions and suspensions, which in turn substantially enabled manipulation of SWNTs into various bulk materials, including films, fibers, solids, and composites of all kinds [23].

### 2.2. Formation of Electrospun PAN Nanofibers and PAN/SWNTs Composite Nanofibers

A 0.75 wt% SWNTs based PAN composite solution was prepared as follows. A given weight of SWNTs was first dispersed for 2 h in DMF through mild bath sonication (KQ-250DB, Kunshan Ultrasonic Instrument Co., Ltd.), which was followed by the addition of PAN (128.91 mg per milliliter of SWNTs/DMF solution). The mixture was then mechanically stirred overnight at 40 °C using a magnetic stirrer to yield a homogeneous solution. For comparison, a pure PAN solution was prepared by a similar method (without the addition of SWNTs).

Then relatively aligned PAN nanofibers and PAN/SWNTs composite nanofibers were obtained by electrospinning. The voltage between the electrode and the counter electrode could be controlled by the high voltage power supply which was set at 14–16 kV. The 0.16 m perimeter collector rotated at a surface speed about 6.6 m/s, that the high speed rotating collector could align the nanofibers into the nanofiber sheets.

### 2.3. Hot-Stretching

During the electrospinning process, however, the whirlpool jet from the pinhead to the collector made it difficult to get unidirectional alignment in a large-area sheet [24]. Therefore, the electrospun nanofibers needed a subsequent hot-stretch to improve the fiber alignment. In this study, the PAN nanofibers and PAN/SWNTs composite nanofibers were hot-stretched according to the method proposed by Phillip and Johnson [25,26]. Both ends of the electrospun PAN/SWNTs sheets were clamped with pieces of graphite plates. Then one end was fixed to the ceiling of the oven and the other end was weighted by a 75 g of metal poise to give a desired tension and elongation in the temperature controlled oven at about 135 °C for 5 minutes.

### 2.4. Morphologies of Nanofibers

The morphologies and diameters of the as-spun and hot-stretched PAN nanofibers and PAN/SWNTs composite nanofibers were observed by scanning electron microscopy (SEM, HITACHI S-4700 FEG-SEM). The diameters of electrospun nanofibers were analyzed with image analyzer software (Image J).

The transmission electron microscopy (transmission electron microscopy (TEM), HITACHI H-800) was used to characterize the orientation of SWNTs in nanofibers, which were directly electrospun on a TEM-copper-grid.

### 2.5. Crystallinities and Orientation of the As-Spun and Hot-Stretched PAN Nanofibers

The crystallinities of the as-spun and hot-stretched PAN nanofiber were investigated with *X*-ray diffractometer (XRD, Rigaku D/max 2500VB2+/PC), operated at 40 kV and 200 mA to produce CuKα radiation (*λ* = 1.54 Å). The percent crystallinity (PC) was obtained by extrapolation of the crystalline and amorphous parts of the diffraction pattern. The crystallite size was calculated by using the formula *L_c_* = *kλ*/(*β*cos*θ*), with *k* = 0.89, *λ* = 1.54 Å, *β* is FWHM, *θ* is Bragg angle [27].

The orientation of the as-spun and hot-stretched PAN nanofibers was also examined by *X*-ray diffractometer (XRD, Bruker D8, Bruker Co). The Herman’s orientation factor,* f*, was determined from the fully corrected azimuthal intensity distribution diffracted from the (*100*) reflection at *d* ≈ 5.30 Å.

(1) $$\begin{array}{cc} f & =[3<\cos^{2}\phi >-1]/2\\ & =\int I\left|\sin\phi \right|[3\cos^{2}\phi -1]/2d\phi /\int I\left|\sin\phi \right|d\phi \end{array}$$

where *φ* is the azimuthal angle between the axis of the molecular segment and of the fiber and *I* is the scattering intensity of the (*100*) reflection at that angle [28].

### 2.6. Thermal Analysis

The *T_g_* of the PAN nanofiber and PAN/SWNTs nanofiber were examined using Differential scanning calorimetry (DSC, METTLER-TOLEDO STARe system). The samples were heated at a scanning rate of 20 °C/min under nitrogen atmosphere in order to diminish oxidation. The value of *T_g_* was found by differentiating the heat flow curve with the temperature.

### 2.7. Mechanical Properties

Mechanical property testing was performed by using a LR30K Electromechanical Universal Testing Machine (LLOYD Company). There were eight specimens used for each nanofiber sample in the tensile test. The samples were prepared in 5 mm width and 20 mm length. The tensile speed in the mechanical test was 20 mm/min. The cross section areas of the samples were calculated via the weights of the samples and the densities of PAN and SWNTs. The average value of the experimental data for each specimen was selected. The tensile strength, tensile modulus, and elongation at break were obtained from the stress-strain curves.

## 3. Results and Discussion

### 3.1. Morphologies

Relatively well-aligned PAN nanofibers were obtained by electrospinning with the drum rotating at high-speeds. However, the whirlpool jet from the pinhead to the collector made it difficult to get much alignment in a large-area sheet, making the subsequent hot-stretched procedure particularly useful.

**Figure 1:**
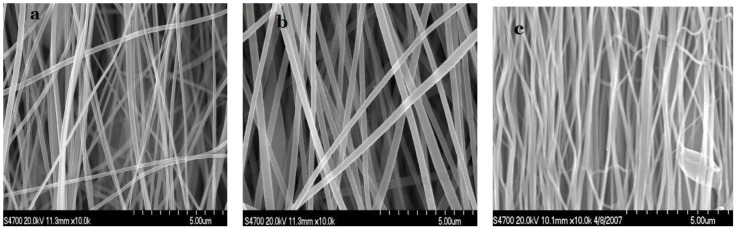
Scanning electron microscopy (SEM) micrographs: (**a**) as-spun pure Partially aligned polyacrylonitrile (PAN) nanofibers; (**b**) PAN/ single-walled carbon nanotubes (SWNTs) composite nanofibers with SWNTs concentration 1 wt%; (**c**) hot-stretched pure PAN nanofibers.

Figure 1 shows SEM micrographs of as-spun and hot-stretched PAN nanofibers and as-spun PAN/SWNTs composite nanofibers respectively. Relatively well-aligned PAN nanofibers were obtained by electrospinning with the cylinder rotating collector at high-speeds to obtain the long and straight nanofibers. From Figure 1(b), it can be seen that there was no obvious conglutination in the nanofibers after the introduction of SWNTs, which proved that SWNTs were relatively dispersed well in the composites. Usually the nanofibers did not align well initially. Figure 1(c) shows the hot-stretched PAN nanofibers, documenting the good alignment along the sheet axis after the hot-stretched process. It can further be found that the alignment of the fibers became closer to parallel after being hot-stretched. Also, the average diameters of the original as-spun fibers were significantly reduced from 173 nm to 115 nm after hot-stretching.

### 3.2. Morphology of SWNTs in PAN/SWNTs Composite Nanofibers

**Figure 2:**
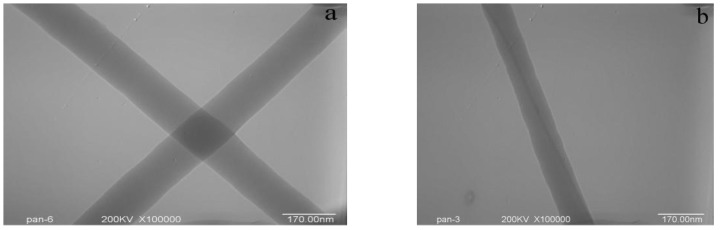

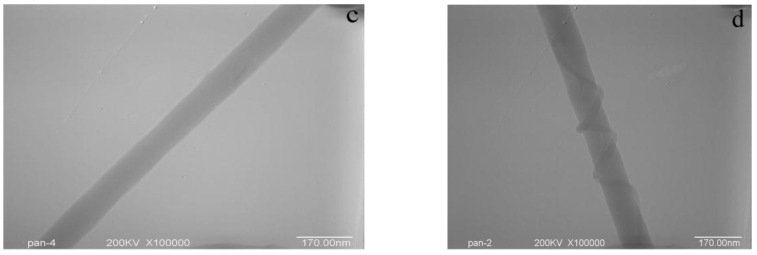
TEM images: (**a**) PAN nanofibers; (**b–d**) PAN/SWNTs nanofibers with SWNTs concentration 1 wt%.

In order to demonstrate that the prepared nanofibers did contain some oriented SWNTs, TEM was utilized to view the alignment and orientation of SWNTs within the nanofibers produced. As seen by TEM in Figure 2, the surface morphology of PAN nanofibers was smooth (Figure 2(a)), but that of the PAN/SWNTs nanofibers was much rougher (Figure 2(b)). Since the SWNTs possess a high electron density compared with the PAN polymer matrix, SWNTs appear as darker tubular structures embedded in the PAN/SWNTs composite nanofibers. It can be seen that the SWNTs were completely wrapped by the PAN matrix. TEM images revealed that in some regions nanotubes oriented well along the fiber axis but the nanotube distribution (number and orientation of the tubes) within a fiber may vary quite significantly (Figure 2(b) and Figure 2(c)). The nanotube distribution within a given fiber was usually quite different from the distribution in others. Topological defects such as entanglements, twisted sections, and knots can at times be observed (Figure 2(d)). It is plausible that the extremely fast electrospinning process, by which carbon nanotubes cannot fully stretch within a millisecond range, leads to such defects.

### 3.3. Crystallinities and Orientation of the As-Spun and Hot-Stretched PAN Nanofibers

**Figure 3:**
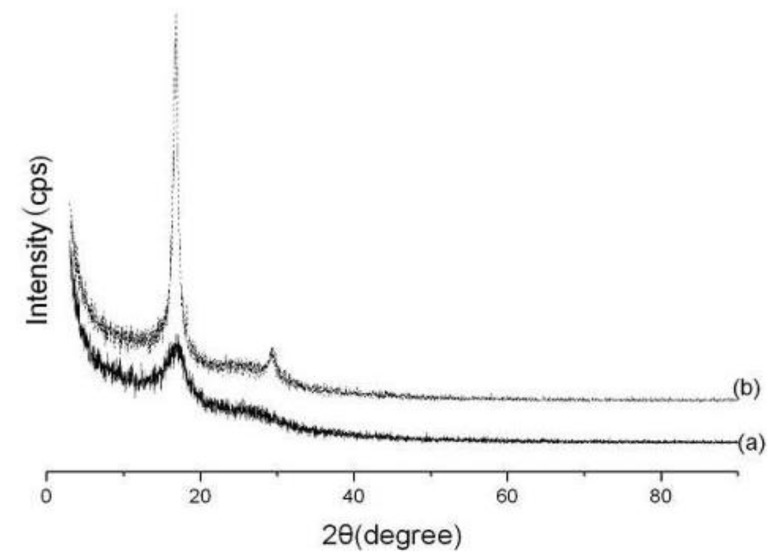
*X*-ray diffraction patterns for the nanofibers: **(a)** as-spun; **(b)** hot-stretched.

Figure 3(a) and (b) show *X*-ray diffraction (XRD) patterns from the as-spun and hot-stretched PAN nanofibers respectively. Noted that the *X*-ray beam is directed perpendicular to normal of the nanofibers, and thus the beam is also parallel to the winding direction of the nanofibers. The nanofibers were highly oriented along the winding direction of the rotating drum collector.

The diffraction pattern showed two equatorial peaks which one was at 2*θ* = 29.5° corresponding to a spacing of *d* ≈ 3.03 Å from the (*110*) reflection and another was at 2*θ* = 17.0° corresponding to a spacing of *d* ≈ 5.30 Å from the (*100*) reflection. The ratio of the *d*-spacing of these two peaks (1.74) is very close to √3:1, indicating hexagonal packing of the rod-like PAN chains [29].

The diffraction pattern of the as-spun nanofiber showed the weak peak with the value of 2*θ* at 17.0°. This indicated that electrospinning of the nanofibers onto a rotating drum generated limited crystallinity. In contrast, the hot-stretched nanofibers showed two diffraction peaks indexed with values of 2*θ* of 17.0° and 29.5°. And it also can be found that the peak at 2*θ* = 29.5° became much bigger after the process of hot-stretched.

Table 1 presents the values of the percent crystallinity, the orientation factor, *f*, the average crystallite sizes for PAN nanofibers. The percent crystallinity of the hot-stretched nanofiber increased about three times in comparison with those of as-spun nanofiber. The orientation factor, *f*, increased from 0.22 to 0.76 after the hot-stretched process. The crystallite size also increased about 162%, indicating highly oriented PAN nanofibers, which was in agreement with the results of the orientation factor [29]. Fibers with larger PAN crystals and higher polymer molecular orientation are expected to lead to a more perfect and higher orientation carbon fiber with improved mechanical properties [30].

**Table 1:** Percent crystallinity and crystallite size obtained from *X*-ray diffraction.

Nanofiber	Crystallinity (%)	Crystallite size(nm)	Orientation factor *f*
As-spun	11.27	4.14	0.22
Hot-stretched	38.34	10.83	0.76

### 3.4. Thermal Behavior

**Figure 4:**
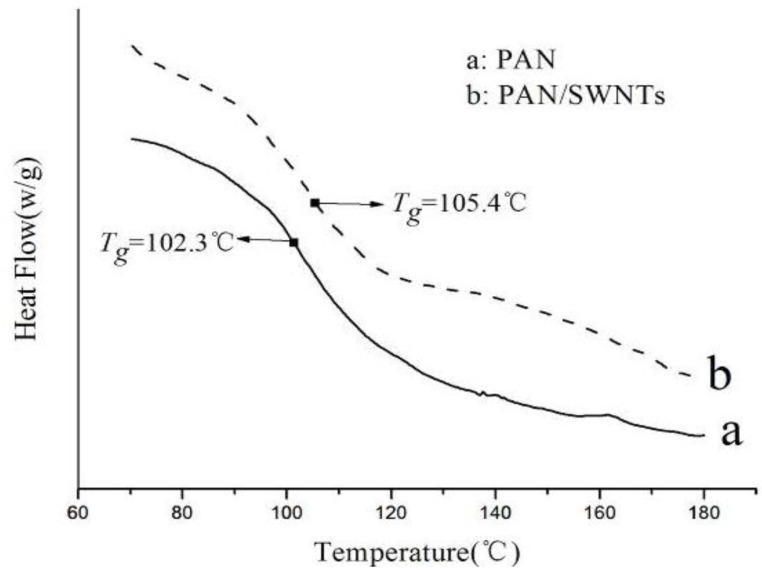
DSC curves of electrospun nanofibers: **(a)** PAN nanofibers; **(b)** PAN/SWNTs composite nanofibers with SWNTs concentration 1 wt%.

Differential scanning calorimetry curves showed the glass transitions (*T_g_)* of PAN and PAN/SWNTs nanofibers, as illustrated in Figure 4. It can be seen that, compared with PAN nanofiber (*T_g_* = 102.3 °C), the *T_g_* was increased by about 3 °C by incorporating only 0.75 wt% SWNTs into the PAN matrix (*T_g_* = 105.4 °C). Increase in the glass transition temperature as compared to the PAN fiber provided evidence of interaction between PAN and SWNTs. The improvement in the *T_g_* stemmed from a stronger interfacial interaction and possible covalent bonding between PAN and the SWNTs. The results suggested that the mobility of PAN chains was reduced due to the constraint effect of SWNTs [31].

### 3.5. Mechanical Properties

In this work, a better representation of the nanocomposites characteristics was attempted by measuring the macroscopic nanofiber sheets. Stretching a piece of the nanofiber sheet gives an assessment of the average mechanical properties of the nanofibers rather than measuring an individual segment of a nanofiber composite [7].

**Figure 5:**
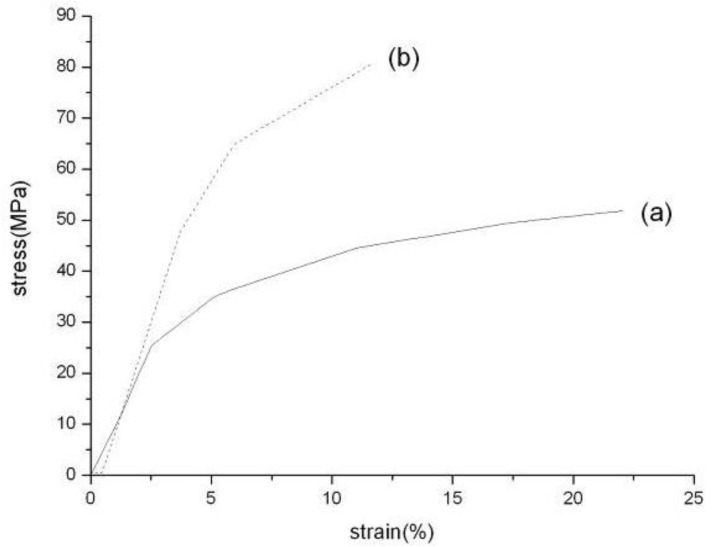
Stress-strain curves of PAN nanofiber sheets before and after hot-stretched: **(a)** as-spun; **(b)** hot-stretched.

The stress-strain curves of PAN nanofibers, before and after hot-stretched respectively, are presented in Figure 5. Hot-stretched improved tensile strength and the modulus of PAN nanofibers. The tensile strength and tensile modulus increased by 55.32% and 156.48% respectively. It can be concluded that the hot-stretched method can improve the mechanical properties of PAN nanofibers. During the process of hot-stretched, the PAN molecular chain moved and arranged again along the fiber axis, the orientation and crystallinity were also improved. Therefore the mechanical properties of PAN nanofibers were improved due to the improvement of orientation and crystallinity. The improvement of crystallite size resulted in the elongation at break decreased obviously. The increased polymer orientation and crystal size point to the potential of PAN/SWNTs composite nanofibers as the precursor for the next generation carbon fiber.

**Figure 6:**
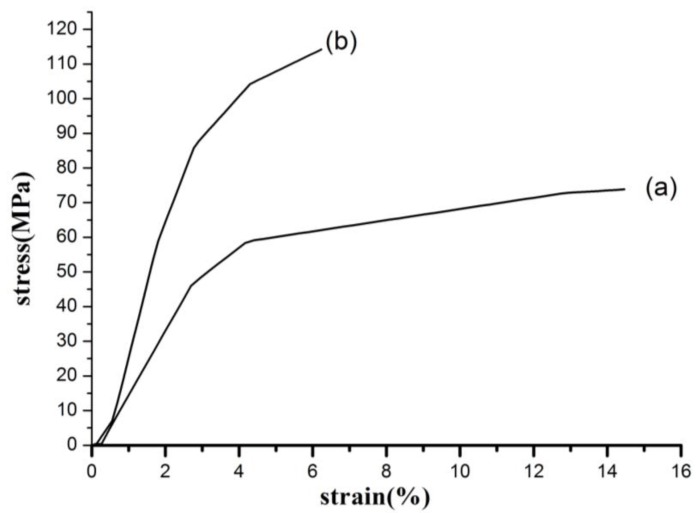
Stress-strain curves of PAN/SWNTs nanofiber sheets with SWNTs concentration 1 wt% before and after being hot-stretched: **(a)** as-spun; **(b)** hot-stretched.

The stress-strain curves of PAN/SWNTs nanofibers, before and after being hot-stretched, are presented in Figure 6. Hot-stretched improved tensile strength and the modulus of PAN/SWNTs nanofibers. The tensile strength and tensile modulus increased by 54.70% and 125.40% respectively. It can be concluded that the hot-stretching can notably improve the mechanical properties of PAN/SWNTs nanofibers.

**Figure 7:**
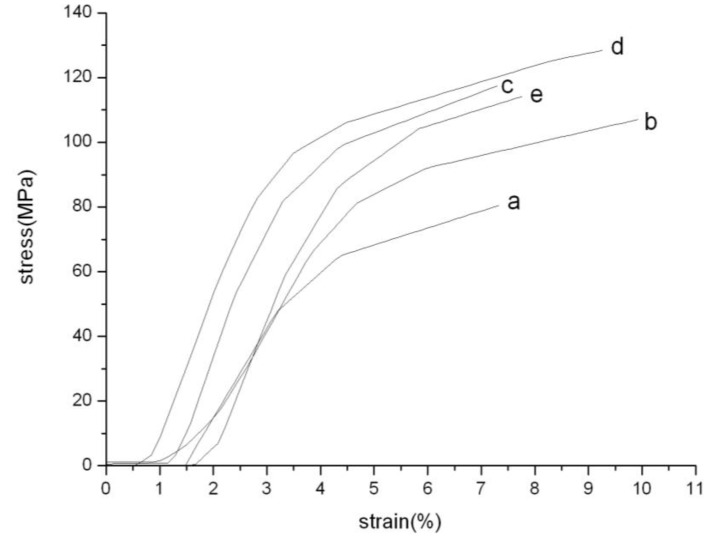
Stress-strain curves for PAN and PAN/SWNTs Nanofiber: (**a**) pure PAN; (**b**) 0.25% SWNTs; (**c**) 0.5% SWNTs; (**d**) 0.75% SWNTs; (**e**) 1% SWNTs.

Figure 7 shows the stress–strain curves of the PAN nanofibers and PAN/SWNTs nanofiber composites after hot-stretching. SWNTs improved the modulus and tensile strength of the nanofiber. The tensile strength 128.76MPa of the nanocomposites at about 0.75% SWNTs by weight was increased with 58.9%. Also the tensile modulus showed a peak value of 4.62GPa with 66.8% improvement. The (e) curve in Figure 7 deviated from the trend, which might be the non-uniform dispersion of SWNTs in high concentration. The significant improvement in strength and modulus is likely related to the good dispersion and orientation of SWNTs within the polymer matrix, and the strong interfacial adhesion due to the SWNTs surface modification [31]. It can be concluded that hot-stretching and the introduction of SWNTs can improve the mechanical properties of PAN nanofibers significantly.

## 4. Conclusions

In this study, PAN nanofibers and PAN/SWNTs composite nanofibers were prepared by electrospinning from PAN/DMF solution. Hot-stretched method was used to increase the degree of crystallinity and molecular orientation of PAN nanofibers and PAN/SWNTs composite nanofibers. Such PAN/SWNTs composite nanofiber sheets represented an important step toward utilizing carbon nanotubes in materials to achieve remarkably enhanced physical properties. An addition of only 0.75 wt% SWNTs to PAN increased the polymer mechanical properties significantly. TEM results showed that SWNTs had a high orientation in PAN/SWNTs composite nanofibers. Compared to pure PAN nanofibers, tensile strength and Young’s modulus of the hot-stretched nanofibers exhibit considerable improvement. The improvement of orientation and crystallinity, the better PAN nanofibers alignment, are all contributed to the obvious increases of mechanical properties of the nanofibers. Thus, the composite nanofibers with the component of SWNTs and the hot-stretched nanofibers can be used as the potential precursor to produce high-performance carbon nanofibers. The mechanical properties of the PAN nanofibers and PAN/SWNTs composite nanofibers can be improved more by extensive studies of electrospinning and the hot-stretching process.
